# Phosphorylation states of cell cycle and DNA repair proteins can be altered by the nsSNPs

**DOI:** 10.1186/1471-2407-5-107

**Published:** 2005-08-19

**Authors:** Sevtap Savas, Hilmi Ozcelik

**Affiliations:** 1 Fred A. Litwin Centre for Cancer Genetics, Samuel Lunenfeld Research Institute, Mount Sinai Hospital, Toronto, M5G 1X5, ON, Canada; 2 Department of Pathology and Laboratory Medicine, Mount Sinai Hospital, Toronto, M5G 1X5, ON, Canada; 3 Department of Laboratory Medicine and Pathobiology, University of Toronto, M5G 1L5, Toronto, ON, Canada

## Abstract

**Background:**

Phosphorylation is a reversible post-translational modification that affects the intrinsic properties of proteins, such as structure and function. Non-synonymous single nucleotide polymorphisms (nsSNPs) result in the substitution of the encoded amino acids and thus are likely to alter the phosphorylation motifs in the proteins.

**Methods:**

In this study, we used the web-based NetPhos tool to predict candidate nsSNPs that either introduce or remove putative phosphorylation sites in proteins that act in DNA repair and cell cycle pathways.

**Results:**

Our results demonstrated that a total of 15 nsSNPs (16.9%) were likely to alter the putative phosphorylation patterns of 14 proteins. Three of these SNPs (CDKN1A-S31R, OGG1-S326C, and XRCC3-T241M) have already found to be associated with altered cancer risk. We believe that this set of nsSNPs constitutes an excellent resource for further molecular and genetic analyses.

**Conclusion:**

The novel systematic approach used in this study will accelerate the understanding of how naturally occurring human SNPs may alter protein function through the modification of phosphorylation mechanisms and contribute to disease susceptibility.

## Background

Phosphorylation is a common, reversible post-translational modification that occurs at serine (S), threonine (T), and tyrosine (Y) residues in proteins [1]. Overall, phosphorylation can alter the structure, function, interaction, stability, and the sub-cellular location of the proteins [2-4], and therefore play an indispensable role in regulation of the cellular processes such as signal transduction, gene expression, cytoskeletal regulation, apoptosis, homeostasis, cell cycle, and DNA damage recognition and repair [5-11]. The phosphorylation state of a protein is determined by the opposing actions of kinases and phosphatases [12]. Proteins may contain multiple phosphorylation sites, which may be targeted by different kinases/phosphatases [2]. The activity of kinases and phosphatases at different times and/or upon different stimuli provides a means of powerful control over the protein phosphorylation state and thus the biological processes the protein is involved in.

In the post-genomic era, there is an expanding interest in identification of the single nucleotide polymorphisms (SNPs) that might affect the protein function and thus contribute to the disease susceptibility. The non-synonymous SNPs (nsSNPs) substitute encoded amino acids in proteins, and therefore are good candidates as disease-modifiers. A variety of approaches have been developed and applied, based on criteria such as the evolutionary conservation status or structural parameters, to characterize and select the nsSNPs that are most likely to have functional consequences [13-19].

In this report, we predicted the potential effect of a set of nsSNPs [20,21] in altering the phosphorylation status of DNA repair and cell cycle proteins using the NetPhos tool [22], which is an artificial neural network method that predicts the phosphorylation sites with a sensitivity of 69–96%. DNA repair and cell cycle pathways interact during the cell growth and division to maintain the genomic stability of dividing cells. Abnormalities in the DNA repair and/or the cell cycle pathways can lead to abnormal cell growth/division or cellular death [23], and are implicated in many human diseases, including cancer [24-30]. Functional significance of many phosphorylated residues of several DNA repair and cell cycle proteins has already been evaluated. For example, phosphorylation of STATα residue S727 is required for its maximal transcriptional activation [31] and enhances its binding to the BRCA1 protein [32]. Similarly, phosphorylation of S383 and S387 are required for the FANCG function during mitosis [33]. Likewise, mutations of the phosphorylated residues Ser366 and Thr387 of p53 affect its transactivation function [34]. To our knowledge, although SNPs of DNA repair and cell cycle proteins have already been shown to contribute to cancer risk [35-37], the potential role of nsSNPs in alteration of phosphorylation patterns of proteins has not been evaluated before. Therefore, the novel approach described in this study will accelerate the formation of a bridge between variations in DNA repair/cell cycle function and predisposition to disease.

## Methods

The nsSNPs extracted from public SNP databases were previously reported [20,21], however, only the nsSNPs that were found in ≥2 chromosomes in a sample panel of ≥46 chromosomes were included into that manuscript. A total of 89 nsSNPs from 47 genes involved in DNA repair and cell cycle constituted the final data set. The NetPhos [22] algorithm was utilized to predict putative phosphorylation sites for both the wild type and the variant protein sequences. Only the predictions that remove or create a site at either the SNP location or at kinase recognition motifs are included into this manuscript. Please note that the BRCA1 and NFKB1 proteins were initially identified as cell cycle protein interacting proteins [21]. However, in this manuscript, we classified the BRCA1 as a DNA repair and the NFKB1 as a cell cycle protein. The mouse orthologues were retrieved from the LocusLink resource of NCBI [38] and aligned with the human proteins using the ClustalW program [39] to identify the corresponding mouse residue.

## Results and discussion

We utilized the NetPhos algorithm to predict putative phosphorylation sites along the DNA repair and cell cycle proteins, and studied whether 89 naturally occurring nsSNPs (64 from 28 DNA repair and 25 from 19 cell cycle genes) might alter the phosphorylation patterns in these proteins. The sensitivity of NetPhos prediction has been reported to be 69–96% with a false-positive prediction rate of 0–26% for Y, 0–11% for S, and 0–14% for T [22]. The results obtained using the NetPhos software are shown in Table I, and are summarized in Table II. Our results have shown that 16.9% (15/89) of the nsSNPs studied are likely to abolish or create 17 putative phosphorylation sites in 44.0% (14/32) of the proteins. As summarized in Table II, five nsSNPs (ERCC5-S311C, OGG1-S326C, XRCC3-T241M, CCND3-S259A, and CDKN1A-S31R) were predicted to abolish putative phosphorylation sites, whereas four nsSNPs were predicted to create putative phosphorylation sites in the proteins (ERCC2-H201Y, ERCC4-P379S, LIG4-P231S, and XRCC1-P309S). These nsSNPs resulted in the addition or removal of a S, T or Y residue at the predicted phosphorylation site.

The kinase recognition/interaction motif involves 7–12 amino acids around the phosphorylated residue [40], and the physicochemical characteristics of these amino acids determine the specificity of the protein kinases [41,42]. Thus, the amino acid substitutions within the kinase recognition motifs are likely to influence the substrate recognition and the subsequent phosphorylation by kinases. Accordingly, we have identified six nsSNPs (Table I, II) located within the phosphorylation motif of six proteins (within 4 amino acids on either side of the putative phosphorylated residue based on NetPhos outputs) that abolished eight putative phosphorylation sites (BRCA1-P871L at S868, BRCA1-S1040N at S1041, ERCC5-S311C at S310, IGHMBP2-T671A at S672, WRN-S1079L at S1083 and at S1084, CCNI-V207I at S208, and NFKB1-H712Q at T716). Interestingly, NetPhos predicts two overlapping phosphorylation motifs for the ERCC5-S311C nsSNP (S311 SLPSSSKMH and S310 ESLPSSSKM), which are both completely abolished by the substitution of the serine residue (position 311) with a cysteine (Table I). Similarly, the WRN-S1079L nsSNP was also predicted to remove 2 putative overlapping phosphorylation motifs (S1083 SKTVSSGTK and S1084 KTVSSGTKE) simultaneously.

The Swiss-Prot [43], HPRD [44], PhosphoBase [45], and Phospho.ELM [46] databases and the existing literature did not reveal any experimentally verified phosphorylation at the predicted sites. Analysis of the mouse orthologues showed that the corresponding amino acids at the BRCA1-S1041, CCNI-S208, ERCC5-S310, IGHMBP2-S672, WRN-S1083 and XRCC3-T241 residues were also predicted to be phosphorylated, suggesting that these motifs/sites might have been evolutionarily conserved between two species. On the other hand, the remaining phosphorylation sites, which are not detected in mouse proteins, may represent the newly evolved phosphorylation motifs in human. However, considering the false-positive rate of NetPhos as well as the possibility that the negative selection acting on the nsSNP sites can result in higher false-positive rates, we cannot totally rule out that all predictions in Table 2 are false. Yet these predictions are still of a great value and suggest possible phosphorylation sites that can be experimentally evaluated. In future, when sufficient molecular data regarding the phosphorylation status of orthologous proteins is available, more systematic analyses can be performed to maximize the accuracy of phosphorylation predictions.

We have also performed an extensive literature review to investigate the role of the reported nsSNPs (minor allele frequencies ≥5%) in human cancer predisposition (Table III). Supporting our hypothesis, three SNPs (CDKN1A-S31R, OGG1-S326C, and XRCC3-T241M) have already found to be associated with altered cancer risk. XRCC3-T241M nsSNP was reported to be associated with increased breast cancer [47,48] and melanoma risk [49], and was also found to be protective against bladder cancer in heavy smokers [50]. XRCC3 is a key DNA repair protein involved in base excision repair [29] and is involved in repairing the alterations caused by many DNA damaging agents. Recently, the XRCC3-M241 variant has been associated with increased risk of incidence of tetraploid cells, frequently observed in cancers, through affecting the function of the XRCC3- and Rad52-associated RPA protein [51]. Similarly, the OGG1-S326C SNP was found to be associated with increased lung [52], orolaryngeal and esophageal cancer risk [53,54]. OGG1 is a DNA repair protein that is protective against the mutations induced by the 8-hydroxyguanine. Yamane *et al*., [55] suggested that OGG1-C326, when compared to OGG1-S326, was associated with a lower repair capacity for 8-hydroxyguanine induced mutations in human cells. In the case of CDKN1A-S31R, the CDKN1A-S31 was suggested to be associated with increased endometrial cancer [56] whereas CDKN1A-R31 was associated with increased primary open-angle glaucoma [57] and esophageal cancer risk [58]. The CDKN1A-R31 form of the protein was not significantly different than the CDKN1A-S31 form in terms of its ability to suppress colony formation [59]. However, it is not clear whether this result would suggest that the CDKN1A-R31 would be functionally equivalent to the wild type allele in other diverse cellular mechanisms that the CDKN1A protein is involved in, such as apoptosis, cell migration, and senescence [60,61].

In addition to the SNPs already implicated in cancer risk, we identified one relatively common nsSNP potentially altering the phosphorylation pattern of a major breast and ovarian cancer susceptibility gene, BRCA1. The BRCA1-P871L SNP was not found to be associated with either breast [62] or ovarian cancer risk [63], however, further analyses is required to see whether this nsSNP or the other nsSNPs in Table III play a role in susceptibility to other cancer types.

How can we explain that commonly occurring nsSNPs (minor allele frequencies ≥5%) are likely to affect the phosphorylation and thus the function of the proteins? If the phosphorylation site is necessary for the function of the protein and the protein is necessary for the fitness of the organism (indispensable/essential protein), then we would expect such nsSNPs (deleterious alleles) to be either removed from the population or be kept at low allele frequency by means of the purifying selection. Thus, in this case, one can conclude that the common nsSNPs presented in this report can be falsely predicted as removing/creating putative phosphorylation sites by NetPhos program. However, the allele frequencies of the deleterious alleles from proteins that are essential for fitness get higher than expected when the nsSNPs are a) created by hot-spot mutation mechanism(s), b) subject to balancing selection, too [64]. Alternatively, even though the nsSNPs (and the abolished/created phosphorylation sites) have important impact on the protein function, the protein and/or the altered protein function may not affect the fitness, which can also explain the lack of purifying selection against such nsSNPs and their relatively high minor allele frequencies. Besides, the biological consequences of altered protein function may only be exerted under certain environmental conditions.

## Conclusion

Here we report a set of nsSNPs in DNA repair and cell cycle genes that are predicted to alter the phosphorylation motifs of the encoded proteins, with possible consequences on protein function, structure, interaction, and stability. If the nsSNPs with a ≥5% minor allele frequency listed in Table III do indeed alter the phosphorylation state of the corresponding proteins, they then represent important candidates for disease susceptibility studies, especially relating to cancer risk. We conclude with the suggestion that our approach and the resulting data indicate a novel mechanism of SNP action: alteration of the functional characteristics of the proteins through phosphorylation may significantly contribute to our understanding of the molecular basis of complex diseases, such as cancer. This study is unique in the sense that it systematically links the possible post-translational modification functional effects of SNPs to disease (cancer) predisposition.

## List of abbreviations

SNP: single nucleotide polymorphism; nsSNP: non-synonymous SNP.

## Competing interests

The author(s) declare that they have no competing interests.

## Authors' contributions

SS participated in design of the study, collected and analyzed the data and prepared the draft of the manuscript. HO participated in the design and coordination of the study, and helped to draft the manuscript. Both authors read and approved the final manuscript.

## Pre-publication history

The pre-publication history for this paper can be accessed here:



## Figures and Tables

**Table 1:** nsSNPs that abolish or create putative phosphorylated residues in DNA repair and cell cycle proteins. Only the NetPhos [22] predictions that remove or create a site at either the SNP location or at kinase recognition motifs are shown. The nsSNPs that create or abolish putative phosphorylation sites at the nsSNP position are shown in bold. Under the wild type and variant columns are the NetPhos outputs with the location of the amino acid, the phosphorylation motif (the putative phosphorylated residue is underlined), the score, and the residue being phosphorylated. 1 and 2 under the frequency column represents the nsSNP minor allele frequencies <5% and ≥5%, respectively [20-21]. Please note that the BRCA1 and NFKB1 proteins were initially identified as cell cycle protein interacting proteins [21]. However, in this manuscript, we classified the BRCA1 as a DNA repair and the NFKB1 as a cell cycle protein. ^§^The putative phosphorylation sites that are also predicted in mouse proteins.

**Pathway**	**Gene**	**Accession #**	**SNP ID**	**nsSNP**	**Freq.**	**Wild Type**	**Variant**
**DNA repair**	BRCA1	NM_007294.1	SNP000007492 rs799917	P871L	2	868 SKRQSFAPF 0.599 *S*	-
	BRCA1	NM_007294.1	rs4986852	S1040N	1	^§^1041 KEASSSNIN 0.557 *S*	-
	ERCC2	NM_000400.1	SNP000000054 rs1799792	H201Y	1	-	201 YSILYANVV 0.745 *Y*
	ERCC4	NM_005236.1	SNP000000067	P379S	1 and 2	-	379 LESNSKWEA 0.507 *S*
	ERCC5	NM_000123.1	SNP001026027 rs2307491	S311C	1	a) 311 SLPSSSKMH 0.990 *S* b) ^§^310 ESLPSSSKM 0.645 *S*	-
	IGHMBP2	NM_002180.1	SNP000012785 rs622082	T671A	2	^§^672 GPATSTRTG 0.634 *S*	-
	LIG4	NM_002312.2	rs3093765	P231S	1	-	231 QLHDSSVGL 0.562 *S*
	OGG1	NM_002542.4	SNP000064679	S326C	2	326 DLRQSRHAQ 0.990 *S*	-
	WRN	NM_000553.2	SNP001026663 rs3087414	S1079L	1	a) ^§^1083 SKTVSSGTK 0.790 *S* b) 1084 KTVSSGTKE 0.829 *S*	-
	XRCC1	NM_006297.1	SNP000064196 rs25491	P309S	1	-	309 EPRRSRAGP 0.996 *S*
	XRCC3	NM_005432.2	SNP000000060	T241M	2	^§^241 SLGATLREL 0.849 *T*	-
**Cell Cycle**	CCND3	NM_001760.2	rs1051130	S259A	2	259 LREASQTSS 0.982 *S*	-
	CCNI	NM_006835.2	rs4252903	V207I	1	^§^208 LAMVSLEME 0.664 *S*	-
	CDKN1A	NM_000389.2	SNP000003435 rs1801270 GAI870831 GAI1503061	S31R	2	31 SEQLSRDCD 0.924 *S*	-
	NFKB1	NM_003998.2	rs4648099	H712Q	1	716 HVDSTTYDG 0.595 *T*	-

**Table 2:** Distribution of the nsSNPs predicted to alter the phosphorylation sites.

**nsSNP**	**DNA repair**	**Cell cycle**	**Total**
Abolished ≥1 putative phosphorylated residue (S, T or Y) at the nsSNP location	3	2	5
Abolished ≥1 putative phosphorylated residue by changing the kinase recognition motif	5	2	7
Created ≥1 putative phosphorylated residue (S, T or Y) at the nsSNP location	4	0	4
Created ≥1 putative phosphorylated residue by changing the kinase recognition motif	0	0	0

**Table 3:** Common nsSNPs with a possible role in cancer predisposition. Only the information derived from the studies on the protein function as well as the studies with a suggestion of disease-association have been included. 1 and 2 under the frequency column represents the nsSNP with minor allele frequencies <5% and ≥5%, respectively [20-21].

**Pathway**	**Gene**	**nsSNP**	**Possible effect on phosphorylation**	**Frequency**	**Functional analysis**	**Cancer risk association**
**DNA repair**	BRCA1	P871L	Abolishes at S869	2	-	-
	ERCC4	P379S	Creates at S379	1 and 2	-	-
	IGHMBP2	T671A	Abolishes at S672	2	-	-
	OGG1	S326C	Abolishes at S326	2	Yamane *et al*. [55]	Sugimura *et al*. [52]; Xing *et al*. [53]; Elahi *et al*. [54]
	XRCC3	T241M	Abolishes and T241	2	Yoshihara *et al*. [51]	Winsey *et al*. [49]; Kuschel *et al*. [47]; Shen *et al*. [50]; Figueiredo *et al*. [48]
**Cell cycle**	CCND3	S259A	Abolishes at S359	2	-	-
	CDKN1A	S31R	Abolishes at S31	2	Chedid *et al*. [59]	Wu *et al*. [58]; Roh *et al*. [56]; Tsai *et al*. [57]
